# T cells form distinct spatially restricted immune responses in synovium of patients with psoriasis arthritis and rheumatoid arthritis

**DOI:** 10.3389/fimmu.2026.1946638

**Published:** 2026-09-30

**Authors:** Nathalie J. Behr, Julio Ramírez, Ana B. Azuaga, Raquel Celis, Gerd Geisslinger, Frank Behrens, Stefano Alivernini, Juan D. Cañete, Klaus Scholich

**Affiliations:** 1 Institute of Clinical Pharmacology, Goethe University Frankfurt, Frankfurt, Germany; 2 Fraunhofer Institute for Translational Medicine and Pharmacology ITMP, Frankfurt, Germany; 3 Fraunhofer Cluster of Excellence for Immune-Mediated Diseases (CIMD), Frankfurt, Germany; 4 Department of Rheumatology, Hospital Clínic and Fundació de Recerca Clinic-Barcelona-Institut d’Investigacions Biomèdiques August Pi i sunyer (IDIBAPS), Barcelona, Spain; 5 Department of Rheumatology, University Hospital Frankfurt, Goethe University Frankfurt, Frankfurt am Main, Germany; 6 Division of Rheumatology and Clinical Immunology, Università Cattolica del Sacro Cuore, Fondazione Policlinico Universitario Agostino Gemelli IRCCS, Rome, Italy; 7 Immunology Core Facility, Gemelli Science and Technology Park, Fondazione Policlinico Universitario Agostino Gemelli IRCCS, Rome, Italy

**Keywords:** dendritic cells, fibroblasts, methotrexate, psoriatic arthritis, rheumatoid arthritis, spatial proteomics, T cells, synovial tissue

## Abstract

**Introduction:**

Psoriatic arthritis (PsA) and rheumatoid arthritis (RA) are chronic, inflammatory joint diseases, causing progressive structural damage. Non-immune cells, such as stromal fibroblasts promote inflammation, cartilage degradation and bone resorption in the synovium. Here, we hypothesized that lining fibroblasts have distinct immune cell neighborhoods in PsA and RA.

**Methods:**

The spatial distribution of structural and immune cell populations was determined using high-multiplex immunohistochemistry in synovial biopsies from 9 PsA and 6 RA patients without previous methotrexate (MTX) treatment and 11 PsA and 11 RA patients treated with MTX treatment.

**Results:**

Dendritic cells and macrophages exhibited strong patient-specific variability, whereas the numbers and localization of T cell subpopulations displayed patterns specific to the disease and treatment. Quantification of overall cell numbers in the synovium revealed a higher Th1/Th2 ratio in PsA than in RA. MTX-treated PsA, patients showed lower Th1 and higher Th2 numbers, whereas in RA, Th1, Th2 and Th9 numbers were less abundant. Spatial analysis revealed two regions with distinct immune responses around the lining fibroblasts. In PsA, the immediate vicinity of the lining fibroblasts was dominated by Th1/Th17 cells, followed by a region with a predominant Th2/Th9 cell population. In RA, a barrier-focused immune response consisting of Th9, Th17, and Th22 cells was observed near the lining fibroblasts. MTX-treated patients had lower the number of Th cells near the lining fibroblasts in PsA but did not differ in Th subtype patterns. In RA, lower Th numbers were mainly observed in the immediate vicinity of the lining fibroblasts, with distinct alterations in the numbers of Th22 and Th2 cells.

**Conclusions:**

In PsA, the immediate vicinity of lining fibroblasts is dominated by Th1/Th17/DC2 cells. In RA, a Th9/Th17/Th22 barrier-centered immune response was present near the lining; Th1 and Th2 cells were essentially absent. Patients treated with MTX had 50% fewer Th cells in this area in both PsA and RA, but increased Th1 numbers in RA. These findings may help explain the different therapeutic responses in these diseases.

## 1 Introduction

Psoriatic arthritis (PsA) and rheumatoid arthritis (RA) are chronic, immune-mediated inflammatory joint diseases, leading to pain, stiffness and progressive structural damage if not treated early (1, 2). RA affects approximately 1% of the global population with a female predominance (3), while PsA has a prevalence of 0.1-0.6%, without gender differences, that strongly correlates with psoriasis, impacting up to 30% of those patients (4). PsA and RA have different genetic, clinical and imaging features, and distinct immuno-inflammatory pathways, but share chronic peripheral joint inflammation characterized by synovial lining hyperplasia, immune cells infiltration and sublining angiogenesis (5). However, several immunohistochemical studies comparing synovial tissue have reported less hyperplasia of the lining layer, a higher density of endothelial cells and a lower number of T cells in PsA compared with RA (6–8). Although antibodies against citrullinated proteins (ACPA) are specific to RA, tertiary lymphoid structures containing T cells and B cells (lymphoid neogenesis) have also been identified in the synovial tissue of patients with PsA (9–11). Methotrexate (MTX) is the first-line conventional synthetic Disease-Modifying Anti-Rheumatic Drug (csDMARD) in both PsA and RA (12, 13). In clinical practice, MTX reduces joint inflammation and structural damage, and improves long-term outcomes; registry data show that MTX retention and escalation patterns are comparable between both diseases (12–14). In synovial tissue, MTX reduces inflammatory infiltrates, including T cells, B-cells and macrophages, as well as proinflammatory cytokines (TNF, IL-1 and IL-6) expression in RA and PsA (15). It also suppresses T cell activation and proliferation, partly by inhibiting folate-dependent pathways and enhancing adenosine-mediated immunosuppression (14, 16).

T cells contribute significantly to the pathogenesis in both diseases, and T cell subpopulations in synovial tissue represent an important proportion of the cellular infiltrate (2, 5). CD4+ T cells can be induced to differentiate into specific helper T (Th) cell subsets, depending on the inflammatory microenvironment: Th1 cells are highly activated in both RA and PsA and promote inflammation through cell-cell interactions, as well as the secretion of cytokines, such as IFNγ, interleukin (IL)-2 and TNF (17). Th2 cells secrete IL-4, IL-5 and IL-13; the cytokines IL-4 and IL-13 typically have an anti-inflammatory effect on type 1 immune responses (18) but can also fulfill pro-inflammatory functions drive and amplify allergic/type 2 inflammation (19). Th9 cells in synovial tissue secrete IL-9 and have pro-inflammatory effects in RA and PsA (20, 21). Th17 cells are similarly present in PsA and RA, but the IL-23/IL-17 pathway has greater clinical impact on the skin and joint in PsA (22, 23). Th22 cells play either a pathogenic or protective role, depending on the stage of the disease, and have been shown to promote osteoclast differentiation through the production of IL-22, thereby playing a key role in bone destruction in patients with RA (24). In addition, IL-22 induces epidermal thickening and remodeling through inhibition of keratinocyte terminal differentiation (25, 26) and mesoderm stem cell proliferation in spondyloarthropathies (27) and may fulfill similar roles in fibroblast proliferation in RA and PsA. A recent study reported correlation between the number of polyfunctional subtypes of Th (Th17, Th1, exTh17) in synovial tissue and disease activity in patients with PsA, highlighting both the translational relevance and the complexity of studying T cell subpopulations (23).

At the single-cell level, PsA and RA exhibit distinct profiles of synovial immune cells, which determine their different clinical and pathological characteristics and justify the different therapeutic targets, although the spatial distribution of these immune cells is not yet fully understood. Therefore, to understand the mechanisms of the disease, it is essential to characterize the spatial organization and cell-cell interactions in the synovial tissue (28). In addition to immune cells, the joint also contains non-immune cells, such as fibroblasts, endothelial cells, mural cells, chondrocytes, osteoblasts and osteoclasts, which can interact directly with one another or with immune cells, or release key mediators that promote inflammation, cartilage degradation and bone resorption (2).

Spatial OMICS approaches showed in the synovium that activated fibroblasts colocalize with immune cell clusters in RA and juvenile idiopathic arthritis, suggesting their functional interactions (29, 30). When this methodology was applied to the synovial tissue of patients with PsA and RA, pro-resolving niches were identified, consisting of CD200^+^ sublining fibroblasts and type 2 innate lymphoid cells, as well as pro-inflammatory niches containing MMP3^+^/IL-6^+^ fibroblasts and inflammatory immune cells in RA, PsA and axial spondyloarthritis (31). A recent study has shown that tissue-resident IL-17-producing CD4^+^/CXCR6^+^ memory cells (TRM17) are the main spontaneous producers of IL-17A in the synovial tissue of spondyloarthropathies (including PsA), and has confirmed their cell-cell interaction with CLEC10A^+^ dendritic cells (DCs) (32). Consequently, the study of the cellular microenvironment surrounding synovial fibroblasts, including T cells, macrophages and dendritic cells, could provide essential information on cell-cell interactions relevant to the pathogenesis of PsA and RA.

Despite its ability to elucidate the states of T cells, such as proliferation and regulation, single-cell RNA sequencing (scRNA-seq) remains incapable of providing a comprehensive characterization of the conventional T cell effector subsets, including Th1, Th2, and Th17 cells, within the context of synovial tissue samples (33). While previous studies studied immunological niches based on the localization of sublining fibroblasts in PsA and RA (29–31, 34), it is unclear whether this occurs in the neighborhood of lining fibroblasts or involves conventional T cell subtypes. Therefore, we aimed to expand previous spatial transcriptomics data by investigating the classical T cell subpopulations with focus on the inflammatory neighborhood of lining fibroblasts in the synovium. In this study, we used high-content multiplex immunohistochemistry with 47 antibodies to study synovial biopsies from PsA and RA cohorts, naïve and treated with MTX, to quantify the immune responses and to determine their spatial immune architectures surrounding lining fibroblasts.

## 2 Methods

### 2.1 Patient recruitment

This retrospective, cross-sectional study was conducted in accordance with the principles of the Declaration of Helsinki. The use of human material was approved by the Ethics Committee of Hospital Clínic de Barcelona (ID: HCB/2020/0100). All participants gave their written informed consent for the use of their samples. The study included 15 without previous csDMARDs treatment (9 PsA and 6 RA), and 22 treated with MTX-treatment (11 PsA and 11 RA). All participants were adult patients from the Arthritis Unit (HCB) who met the CASPAR criteria for PsA (35) or the ACR/EULAR-2010 criteria for RA (36), with at least one inflamed knee joint (**Table 1**). Patients with untreated PsA had mainly swollen knee joints and patients with MTX treatment had active mono- or oligo-arthritis. Patients with RA, both those untreated and those with an inadequate response to MTX, presented an oligoarticular phenotype, which included knee inflammation. In both groups, indication of rheumatology arthroscopy was therapeutic, with joint lavage and further local corticosteroids administration. All patients of the MTX group were treated with MTX and continued with active monoarthritis or oligoarthritis and low or moderate disease activity (**Table 1**). Previous or current use of targeted therapies was an exclusion criterion. Synovial tissue biopsies were obtained by rheumatological arthroscopy, performed under diagnostic and/or therapeutic indications (lavage) with a 2.7 mm arthroscope (Storz, Tullingen, Germany) (9) and snap-frozen.

**Table 1:** Patient characteristics.

Demographic and clinical data	PsA, - MTX	PsA, + MTX	RA, - MTX	RA, + MTX
N	9	11	6	11
Age, median (IQR)	66.50 (62.00-72.00)	54 (48.60-62.20)	57.50 (52.50-64-40)	67.00 (55.00-71.00)
Female, n (%)	3 (33.3)	4 (36.4)	3 (50)	6 (54.5)
BMI, median (IQR)	27.00 (26.70-28.40)	28.23 (25.35-29.60)	26.03 (23.21-29.38)	25.67 (24.36-29.62)
Treatment Duration (month), median (IQR)	–	37.50 (17.25-48.00)	–	46 (13–96)
Years evolution disease, median (IQR)	3.65 (1.60-10.90)	7.85 (3.08-33.53)	7.65 (2.15-22.75)	8.60 (3.30-14.00)
Erosive disease, n (%)	4 (50)	8 (72.7)	6 (85.7)	7 (100)
TJC, median (IQR)	1.00 (1.00-2.00)	1 (1.00-2.00)	1.50 (1.00-5.00)	1.00 (1.00-3.00)
SJC, median (IQR)	1.00 (1.00-1.00)	1 (1.00-2.00)	1.50 (1.00-5.00)	2.00 (1.00-3.00)
CRP, median (IQR)	2.05 (0.76-2.61)	0.64 (0.23-2.23)	2.85 (1.44-3.00)	1.70 (0.87-7.80)
ESR, median (IQR)	12.00 (9.00-35.00)	10 (6.50-23.50)	15.00 (8.00-27.00)	23.50 (17.00-30.00)
DAS28 CRP, median (IQR)	3.34 (2.93-3.70)	2.87 (2.54-3.68)	3.60 (3.43-4.02)	3.61 (2.97-4.28)
DAS28 ESR, median (IQR)	3.54 (2.81-4.61)	3.10 (2.43-3.44)	3.35 (2.83-4.42)	3.53 (3.33-4.91)
ACPA+ (%)	0	0	83	82
RF+ (%)	0	0	100	73

ACPA, anti-citrullinated protein-antibodies; RF, rheumatoid factor.

### 2.2 Krenn-synovitis-score hematoxylin-eosin staining

Frozen sections were fixed in 4% paraformaldehyd (PFA; Carl Roth, Karlsruhe, Germany), rinsed, and Hematoxylin-Eosin (H&E) stained (Sigma-Aldrich, Taufkirchen, Germany) according to standard histological procedures. Sections were dehydrated, cleared, and mounted for imaging (Keyence BZ-X810). Synovitis severity was graded according to three synovial tissue features (synovial lining cell layer, stromal cell density and inflammatory infiltrate respectively), each ranked on a scale from none (0), slight (1) and moderate (2) to strong (3). Biopsies showing no clear lining layers were not included in the final scoring. The analysis was done manually and included the assessment of the whole tissue sections and the highest score obtained from its analysis was recorded. The values of the parameters were summed and interpreted as follows: 0–1=no synovitis; 2–4=low-grade synovitis; and 5–9=high-grade synovitis (37). The total synovitis score was assessed by a single evaluator blinded to all clinical and demographic data. Data were subsequently combined and analyzed after completion of individual data collection.

### 2.3 Antibody-based spatial proteomics

Frozen tissue was embedded in Tissue-Tek-O.C.T. (Sakura Finetek, Umkirch, Germany) and sections with a thickness of 8 μm were prepared using a cryostat (Leica CM3050S, Leica Biosystems, Wetzlar, Germany) with SuperFrostPlus microscope slides (VWR International GmbH, Darmstadt, Germany). Slides were mounted onto a MACSwell Imaging Frame (Miltenyi Biotec, Bergisch Gladbach, Germany), fixed with 4% PFA/PBS (Carl Roth, Karlsruhe, Germany) and blocked with 3% bovine serum albumin/PBS (Carl Roth, Karlsruhe, Germany). The MACSima Imaging System was used for antibody-based spatial proteomics with a 20x (NA 0.45) objective (Miltenyi Biotec, Bergisch Gladbach, Germany). Briefly, before addition of antibodies an image of the autofluorescence was taken for all channels. Then the sample was incubated with fluorescence-labelled antibodies and washed with PBS to remove unbound antibodies. Then the fluorescence images were taken and the fluorescence was bleached. Afterwards the process was repeated for the next antibodies. For data analysis, the post-bleaching images were subtracted from their following fluorescence image. All antibodies are listed in the **Supplementary Table S1** and representative images are shown in (**Supplementary Figure S2**). For each patient all tissue areas with a minimal size of 560 x 460 µm were measured using at least four serial tissue slices (1–3 areas/tissue sample).

### 2.4 Image analysis

Individual raw FoV images were processed using the automated analysis pipeline implemented in the MACS iQ View software (Version 1.3.1; Miltenyi Biotec, Bergisch Gladbach, Germany). Advanced cell segmentation was performed using nuclear detection based on DAPI staining. Parameters selected were 18–60 pixels for min/max diameter, 100% for detection sensitivity, 80% for separation force, and 1.0 for smoothing filter sigma. Cytoplasmic signal was assigned to single cells by using the “Donut” option with a donut width of 15 pixels. Marker expression was allocated to the segmented cells and used for gating of cell populations and cell type annotation. Marker expression was allocated to the segmented cells and used for gating of cell populations and cell type annotation. Automated gating threshold settings were evaluated with the corresponding staining images. (Sub-)gating of cell populations were adjusted for ROI and cell type-specific cell expression patterns, visualized as scatter plots and histograms within MACS iQ View. The main marker for the identification of the immune cell types are listed in **Table 2**. Cell identification was done by subsequent gating for endothelial cells (CD31), followed by sublining (CD90), lining (CD55), T cells (CD3), neutrophils (CD66b), dendritic cells (CD11c), plasma cells (CD138), mast cells (CD117), natural killer (NK) cells (CD56), macrophages (CD14) and B cells (CD19). Major subtypes for T cells were Th (CD4), cytotoxic T cells (Tc) (CD8a) and NKT cells (CD161). A list with the most relevant markers these cells and other subtypes (Tcm, Th1, Th2, Th9, Th17 and Th22) are shown in **Table 2**. Neighborhood analysis of annotated cell types (i.e. lining fibroblasts) was performed with the MACS iQ View distance analysis tool. Based on the generated distance histograms, ranges of 20 µm (118 pixels) or 60 µm (353 pixels) surrounding the cell type of interest were defined. The experimenter was unaware of the groupings during the analysis. Combined data analysis was performed after the collection of the individual data was complete.

**Table 2:** Markers used to identify cell populations.

Immune cell type	Marker				
Endothelial cells	CD31^+^				
Sublining	CD90^+^				
Lining	CD55^+^				
T cells	CD3^+^				
Neutrophils	CD66b^+^				
Dendritic cells	CD11c^+^				
Plasma cells	CD138^+^				
Mast cells	CD117^+^				
NK cells	CD56^+^				
Macrophages	CD14^+^				
B cells	CD19^+^				
Cytotoxic T cell	CD3^+^	CD8^+^			
NKT cell	CD3^+^	CD56^+^	CD161^+^		
Th1	CD3^+^	CD4^+^	CCR6^-^	CCR3^-^	CCR5^+^
Th2	CD3^+^	CD4^+^	CCR6^-^	CCR3^+^	CCR4^+^
Th9	CD3^+^	CD4^+^	CCR6^+^	CCR4^-^	
Th17	CD3^+^	CD4^+^	CCR6^+^	CCR4^+^	CD161^+^
Th22	CD3^+^	CD4^+^	CCR6^+^	CCR4^+^	CD161^-^
Threg/Undefined Th cell	CD3^+^	CD4^+^			

### 2.5 Statistical analysis

For each of the 37 patients, at least four tissue sections, each containing one to three regions of interest, were analyzed and averaged to generate a single value per patient. Data are presented as mean ± SEM. For multiple group comparisons, two-way ANOVA was performed followed by Tukey’s or Holm-Šídák *post-hoc* tests. Pairwise comparisons were assessed using an unpaired two-tailed Student’s t-test with Holm-Šídák correction. Normality of data distribution was assessed using the Shapiro-Wilk test, and homogeneity of variance was assessed by examining residual plots. Statistical analyses were conducted in GraphPad Prism v9.0.1, with p < 0.05 considered significant.

## 3 Results

### 3.1 Study design and tissue harmonization

This study included a total of 37 patients: 15 without previous csDMARDs treatment (9 PsA and 6 RA), and 22 treated with MTX (11 PsA and 11 RA) (**Figure 1A**). Demographic and clinical characteristics of the patients are detailed in **Table 1**. Median disease activity based on DAS28-CRP score was within the low disease activity for PsA and RA patients non-treated or treated with MTX (**Table 1**). The Krenn-Synovitis-Score (KSS) was not significantly different between non-treated patients and those treated with methotrexate, but was higher in RA than in PsA patients. This difference was solely due to the higher lining score in RA, as immune infiltrates and stromal cells were similar in all patient groups (**Supplementary Figure S3**). High content imaging analysis using antibody-based spatial proteomics (38, 39) was employed to determine disease- and treatment-specific differences in the content and the localization of the immune cells in the biopsies. The biopsies were subjected to high-content imaging using 47 antibodies (**Supplementary Figures S1, S2**), which allowed the identification of relevant immune and non-immune cells as well as their subtypes (38). For bioinformatics analysis, a segmentation mask was generated based on nuclear staining and was used to extract single-cell expression data from the images of all markers (**Figure 1B**). Single-cell phenotyping was followed by the identification of cell clusters representing different immune cell types and cell-cell distance mapping (**Figure 1C**). For clarity, only the main markers used to identify a specific cell type will be mentioned in the text. To harmonize the tissue samples, tissues with rare features, such as very high lipid accumulation (n = 1), necrotic areas with low cell counts (n = 5) as well as large arterioles or venules (n = 2), were omitted from the analysis. This reduced the original number from 45 biopsies to 37 biopsies used in the analysis. As result, similar amounts of endothelial cells (CD31^+^), lining (CD55) and sublining (CD90) and immune cells (CD45^+^), were observed in the tissue samples (**Figures 1D, E**).

**Figure 1:**
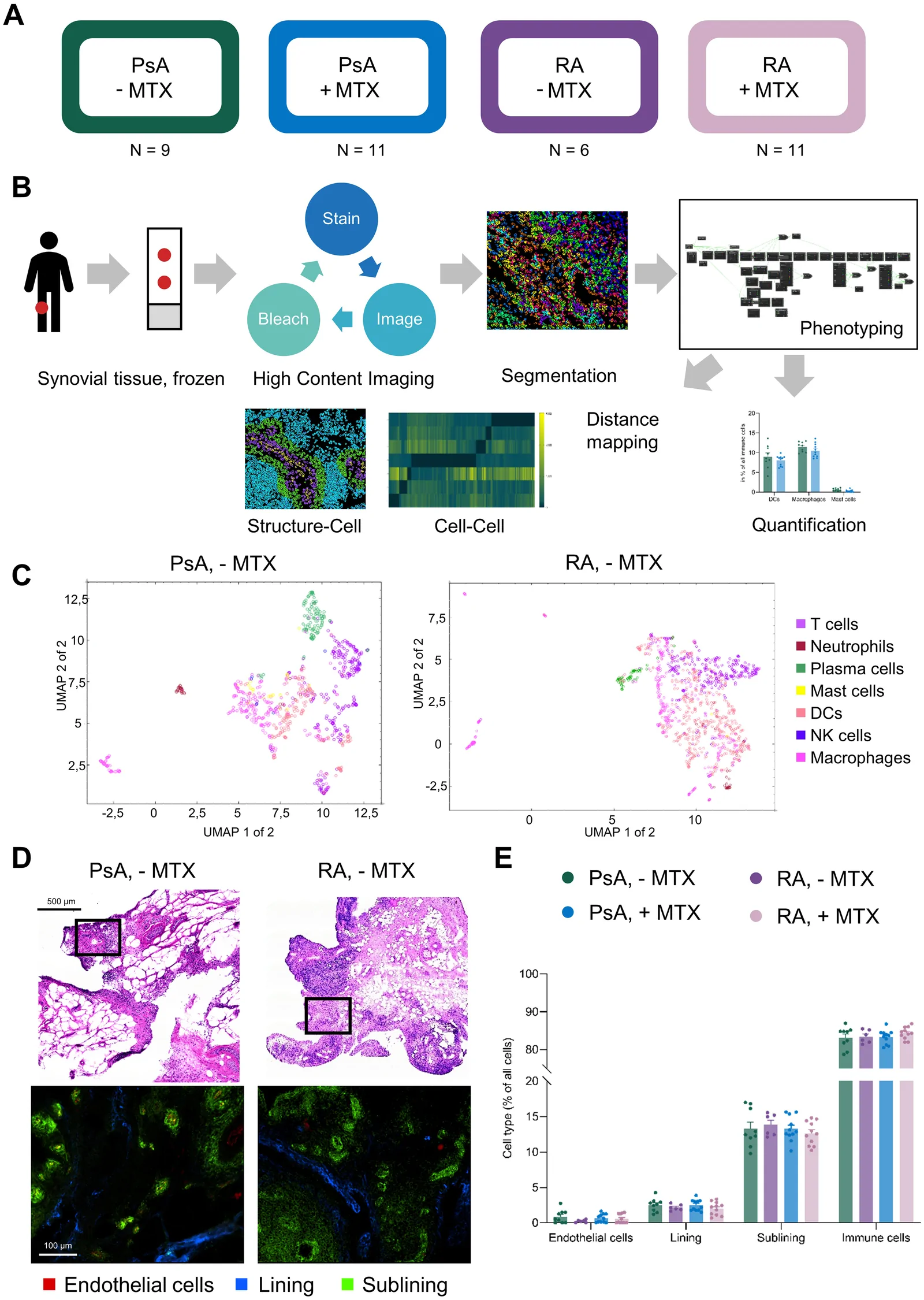
Spatial proteomics workflow of synovial biopsies from PsA and RA patients with or without methotrexate treatment. **(A)** Clinical cohorts included in the study, comprising PsA and RA patients who are either treatment naïve or receiving methotrexate. **(B)** Experimental and bioinformatic workflow: Frozen synovial tissue sections are prepared on slides and subjected to repeated cycles of staining, imaging, and signal erasure to achieve highly multiplexed high‐content imaging. **(C)** Representative UMAP image showing the clusters for the main immune cell types in PsA and RA patients without MTX treatment. **(D)** Representative images showing H&E and immunofluorescence staining of synovial tissue from PsA and RA patients without MTX treatment, with endothelial cells (red), lining (blue), and sublining (green) regions; scale bar: 100 µm. **(E)** Graph showing the number of endothelial cells (CD31), immune cells (CD45) as well as lining (CD55) and sublining (CD90) for PsA (N = 9) and RA (N = 6) patients without MTX treatment, as well as PsA (N = 11) and RA (N = 11) patients with MTX treatment. Two-way ANOVA with *post-hoc* Tukey’s multiple comparison test.

### 3.2 DCs and NKT cells, but not macrophages and Tc cells, show disease- and treatment-specific quantitative alterations

In a first step, we determined the overall numbers of the major identified immune cell types together with their most abundant subtypes. In all patient groups, the most abundant major immune cell types were macrophages, DCs, and T cells, with significantly higher numbers of DCs in RA without MTX treatment as compared to PsA (**Figure 2A**). In PsA, the numbers of the major immune cell types did not differ significantly between patients with or without MTX, whereas in RA, the number of DCs was nearly 50% lower (**Figures 2B, C**). A detailed analysis of DC subpopulations revealed that the majority of DCs were CD11c^+^/HLADR^+^ DC2, whereas the CD11c^+^/HLADR^-^ DC populations were highly heterogeneous and present in only a subset of patients (**Figures 2D, E**). In the PsA patient group receiving MTX treatment, the numbers of the various DC subpopulations were similar to those in the group not receiving MTX treatment (**Figures 2F, G**). In contrast, MTX treatment of RA patients did not affect the DC2 population, but significantly reduced the number of DCs within the CD11c^+^/HLADR- population (**Figures 2F, G**).

**Figure 2:**
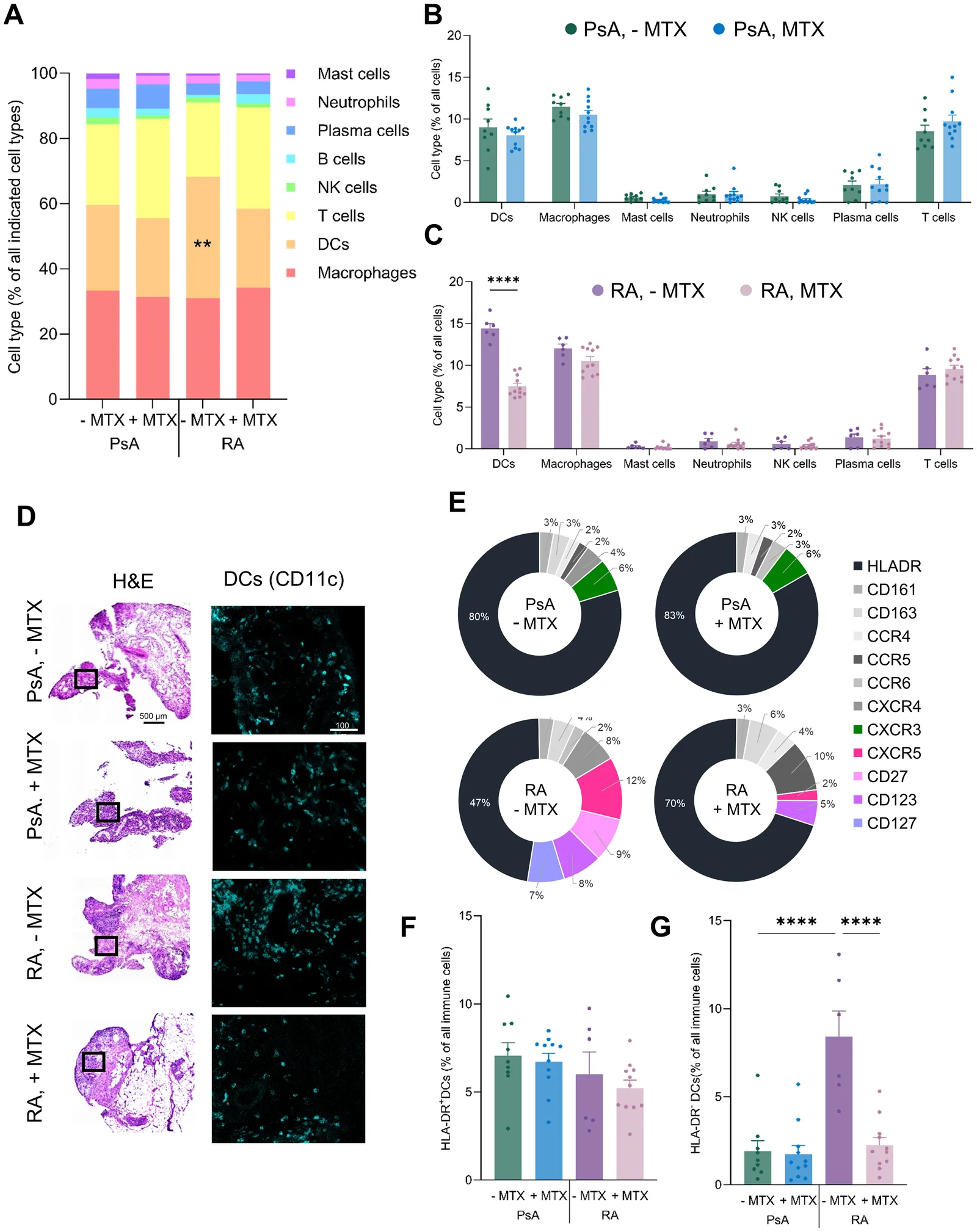
Overview of synovial immune cell composition in PsA versus RA patients. **(A)** Percentage of major immune cell populations (macrophages, mast cells, dendritic cells, plasma cells, NK cells, T cells, and neutrophils) in synovial tissues from the PsA and RA patients. Data are shown as mean of N = 6-11. Multiple unpaired t-test with *post-hoc* Holm-Šídák multiple comparison test, **p<0.01: PsA vs RA -MTX. **(B, C)** Percentages of immune cell subsets from PsA **(B)** and RA **(C)** patients with or without MTX treatment. Data are shown as mean of N = 6-11. Multiple unpaired t-test with *post-hoc* Holm-Šídák multiple comparison test, ****p<0.0001. **(D)** Representative H&E stainings and immunofluorescence images of CD11c^+^ DCs in synovial tissues from untreated and MTX-treated PsA and RA patients; scale bar: 100 µm. **(E)** Percentage of DC subsets among all CD11c^+^ cells in untreated and MTX-treated PsA and RA synovial tissues. Data are shown as mean of N = 6-11. **(F, G)** Percentage of the number of HLADR^+^ **(F)** and HLARD^-^ DCs **(G)** is shown. Two-way ANOVA with *post-hoc* Tukey multiple comparison test, ****p<0.0001.

A similar patient-specific subtype heterogeneity was observed for macrophages where again one dominant subpopulation (CD14^+^/CD206^+^/CD163^-^) prevailed (**Figure 3A**). MTX-treated patients did not differ significantly in the number of dominant CD14^+^/CD206^+^/CD163^-^ cells or the CD14^+^/CD206^-^ populations in PsA and RA (**Figures 3B, C**). Three main types of T cells were observed in patients with PsA and RA. Th cells were the most abundant in both disease groups without MTX treatment, followed by Tc and NKT cells (**Figure 3D**). Synovial tissue from patients treated with MTX showed a lower percentage of Th cells in relation to Tc and NKT cells in both diseases (**Figure 3D**). The number of Tc cells (CD3+/CD8+) was nearly identical in patients with PsA and RA, but higher in both diseases during MTX treatment (**Figures 3E, F**). The higher Tc numbers in PsA were based on increased formation of memory Tc (Tcm) cells (**Figure 3G**). In untreated patients, 55% of all Tc cells were Tcm cells, whereas in MTX-treated patients, around 75% of all Tc cells were Tcm cells. In contrast, the number of Tc and Tcm cells increased proportionally in RA, with Tcm cells comprising around 60% of all Tc cells (**Figure 3G**). Unlike Tc cells, NKT cells were less abundant in PsA patients than in RA patients without MTX treatment. There were four times more NKT cells in RA patients (**Figures 3H, I**). Patients treated with MTX had even lower numbers of NKT cells in PsA, while the number of NKT cells nearly doubled in RA patients (**Figure 3I**). This difference was confirmed by stratifying the cohort based on synovitis degree, as low-grade synovial inflammation in RA patients is enriched in NKT cells compared to PsA patients (**Supplementary Figure S4**).

**Figure 3:**
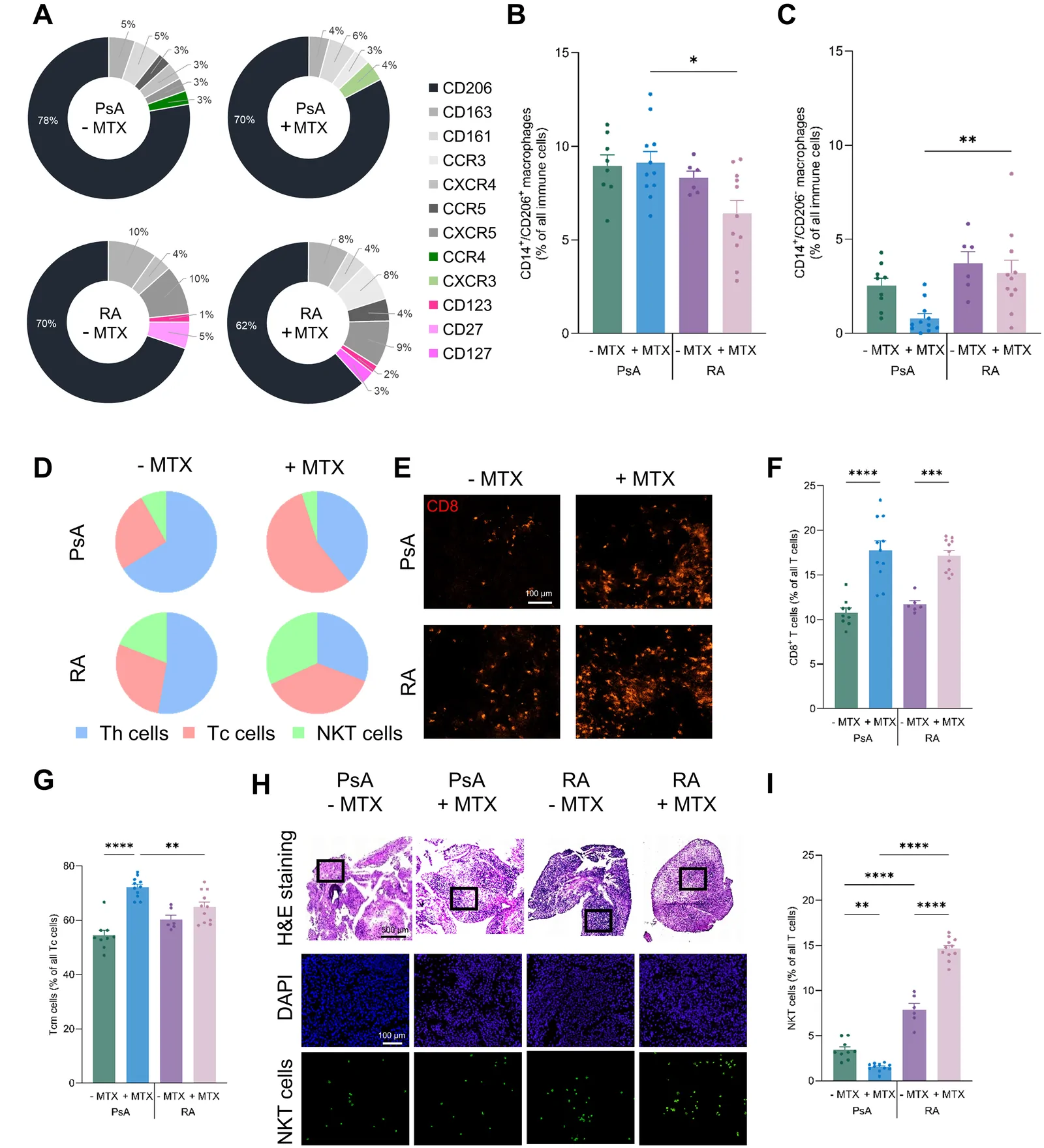
Cytotoxic T cells and NKT cells show distinct responses to MTX treatment in PsA and RA patients. **(A)** Proportions of macrophage subsets among CD206^+^ cells in synovial biopsies. Data are % of all macrophages (N = 6-11). Disease-specific subpopulations are shown in green for PsA and pink for RA. **(B, C)** Number of CD14^+^/CD206^+^ **(B)** and CD14^+^/CD206^-^ **(C)** macrophages. Data are mean of N = 6-11. Two-way ANOVA with *post-hoc* Tukey multiple comparison test, *p<0.05, **p<0.01. **(D)** Percentage of T cell subtypes. Data are % of all immune cells. **(E)** Representative images showing CD8^+^ Tc cells in synovial tissues. Scale bar: 100 µm. **(F, G)** Percentage of Tc cells of all T cells **(F)** and percentage of Tcm of all Tc cells **(G)**. Data are mean of N = 6-11. Two-way ANOVA with *post-hoc* Tukey multiple comparison test, **p<0.01, ***p<0.001, ****p<0.0001. **(H)** Representative H&E stainings and immunofluorescence images of NKT cells (green); scale bar: 100 µm. **(I)** Percentages of NKT cells of all T cells. Two-way ANOVA with *post-hoc* Tukey multiple comparison test, **p<0.01, ****p<0.0001.

Taken together, the observed disease-specific differences for NKT cells and DCs underscore the distinct immune responses that drive both diseases. Meanwhile, the heterogeneity of DC and macrophage subpopulations highlights the flexible responses of these cell types to patient-specific conditions.

### 3.3 Th cell responses differ between PsA and RA especially during MTX treatment

The number of Th cells (CD3^+^/CD4^+^) was significantly higher in PsA than in RA in the observed areas, while in both diseases their number was lower in patients receiving MTX treatment (**Figure 4A**). Accordingly, the CD4/CD8 T cell ratio in the synovium was 2.7 in PsA and 1.9 in RA, while being < 1 for MTX-treated patients in both diseases (**Figure 4B**). A detailed analysis of Th cell populations showed a higher number of Th1 cells and a lower number of Th9 cells in untreated PsA than in untreated RA, while no significant differences were observed in Th2, Th17, and Th22 cells (**Figure 4C**). MTX-treated patients showed several disease-specific differences affecting all Th cell subpopulations, except Th22 cells. In PsA, lower numbers of Th1 and Th17 cells together with higher Th2 cell numbers were seen (**Figure 4D**). In contrast, in RA the MTX-treated patients had lower Th1, Th2 and Th9, but higher Th17 numbers (**Figures 4E, F**). Taken together, MTX-treated patients had in PsA a shift of the immune response from the more destructive Th1 to Th2 cells whereas in RA, MTX treatment did not alter the Th1/Th2 ratio (**Figures 4F, G**). Principal component analysis of all cell type numbers did not distinguish between PsA and RA patients, but showed a clear difference between patients with and without MTX treatment (**Figure 4H**; **Supplementary Figure S5**).

**Figure 4:**
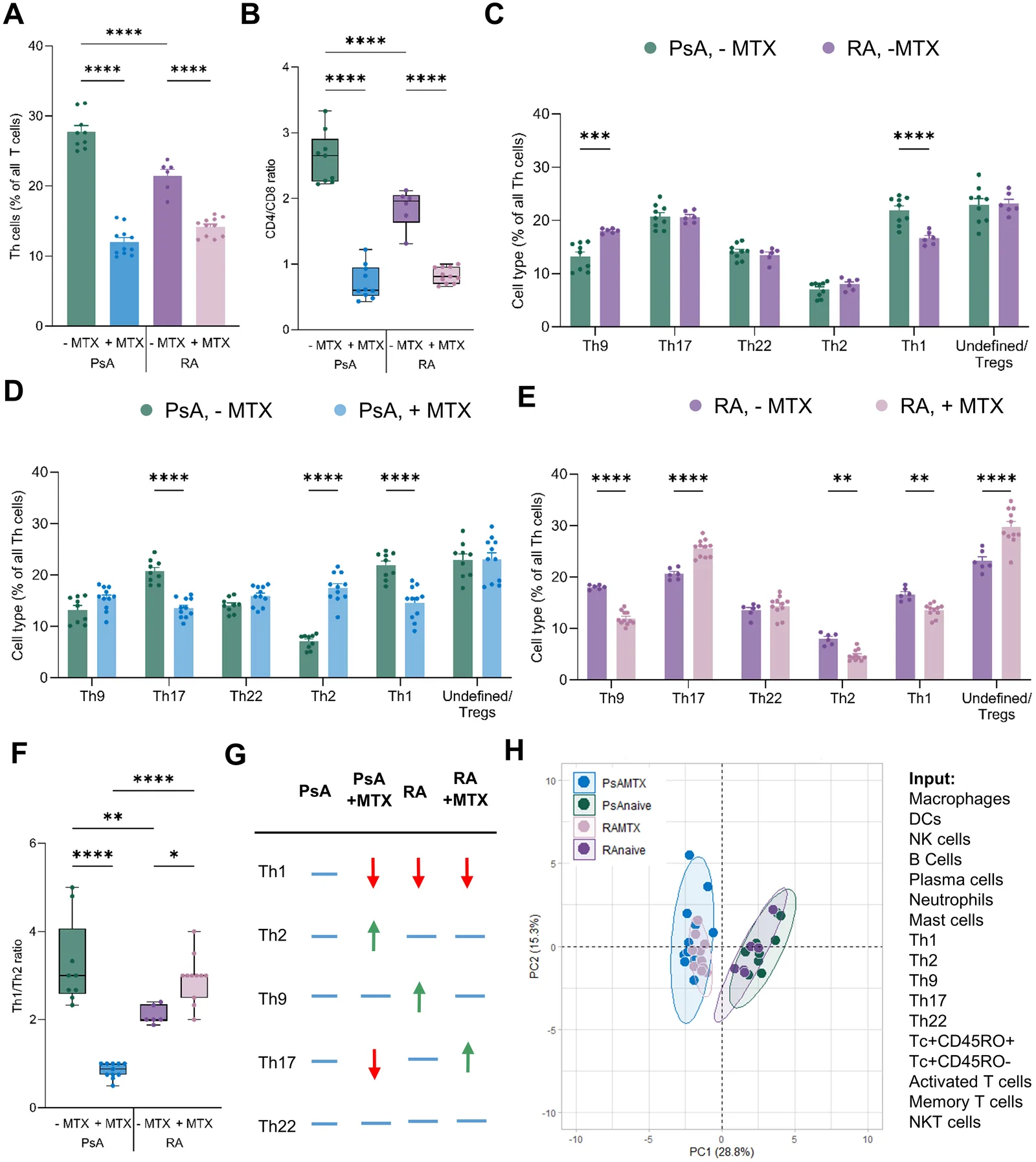
MTX treatment leads to reduction in T helper cells in PsA and RA patients. **(A)** Percentages of Th cells among total T cells in synovial tissues. Data are mean of N = 6-11. Two-way ANOVA with *post-hoc* Tukey multiple comparison test, ****p<0.0001 **(B)** Ratio of CD4^+^ and CD8^+^ T cells in the biopsies. Two-way ANOVA with *post-hoc* Tukey multiple comparison test, ****p<0.0001 **(C)** Percentage of Th cells of all T cells. Multiple unpaired t-test, with *post-hoc* Holm-Šídák multiple comparison test, ***p<0.001, ****p<0.0001. **(D, E)** Percentage of Th cell of all T cells in synovial tissues from PsA **(D)** and RA **(E)** patients. Multiple unpaired t-test, with *post-hoc* Holm-Šídák multiple comparison test, **p<0.01, ****p<0.0001. **(F)** Ratio of Th1 and Th2 cells of patients without and with MTX-treatment PsA (green/blue) and RA (purple/pink). Two-way ANOVA with *post-hoc* Tukey multiple comparison test, *p<0.05, **p<0.01, ****p<0.0001. **(G)** The numbers of Th1, Th2, Th9, Th17, and Th22 cells were compared across the four patient groups, with untreated PsA serving as the reference group. Arrows indicate significant differences to the PsA group. **(H)** Principal component analysis based on the indicated immune cell numbers in untreated PsA (N = 9) and RA (N = 6) patients, as well as in MTX treated PsA (N = 11) and RA (N = 11) patients.

### 3.4 Spatially restricted distinct Th cell immune responses near the synovial lining

Since the various Th cell subtypes, for example Th1 and Th2 cells, mediate very different immune responses, we studied whether the Th cell populations are separated into specific immunological niches. Therefore, we performed a distance mapping using lining fibroblasts (CD55^+^) staining as starting point to determine for all immune cells the number of cells in the direct neighborhood of the lining fibroblasts (0-20 µm distance), in their wider proximity (21-60 µm) and in greater distance (>60 µm) (**Figure 5A**). We found that the DC2 population was evenly distributed between all three regions in untreated PsA while in RA they had a higher density in greater distance (>60 µm) to the lining fibroblasts independently of MTX treatment (**Figures 5B, C**). Macrophages and NKT cells were distributed with even densities in all three regions whereby MTX-treated PsA showed higher macrophage density in the direct neighborhood of the lining fibroblasts (**Figures 5D–G**). Also, Tc cells were evenly distributed in both diseases, but MTX-refractory patients showed doubled Tc density in the two areas next to the lining fibroblasts (**Figures 5H, I**).

**Figure 5:**
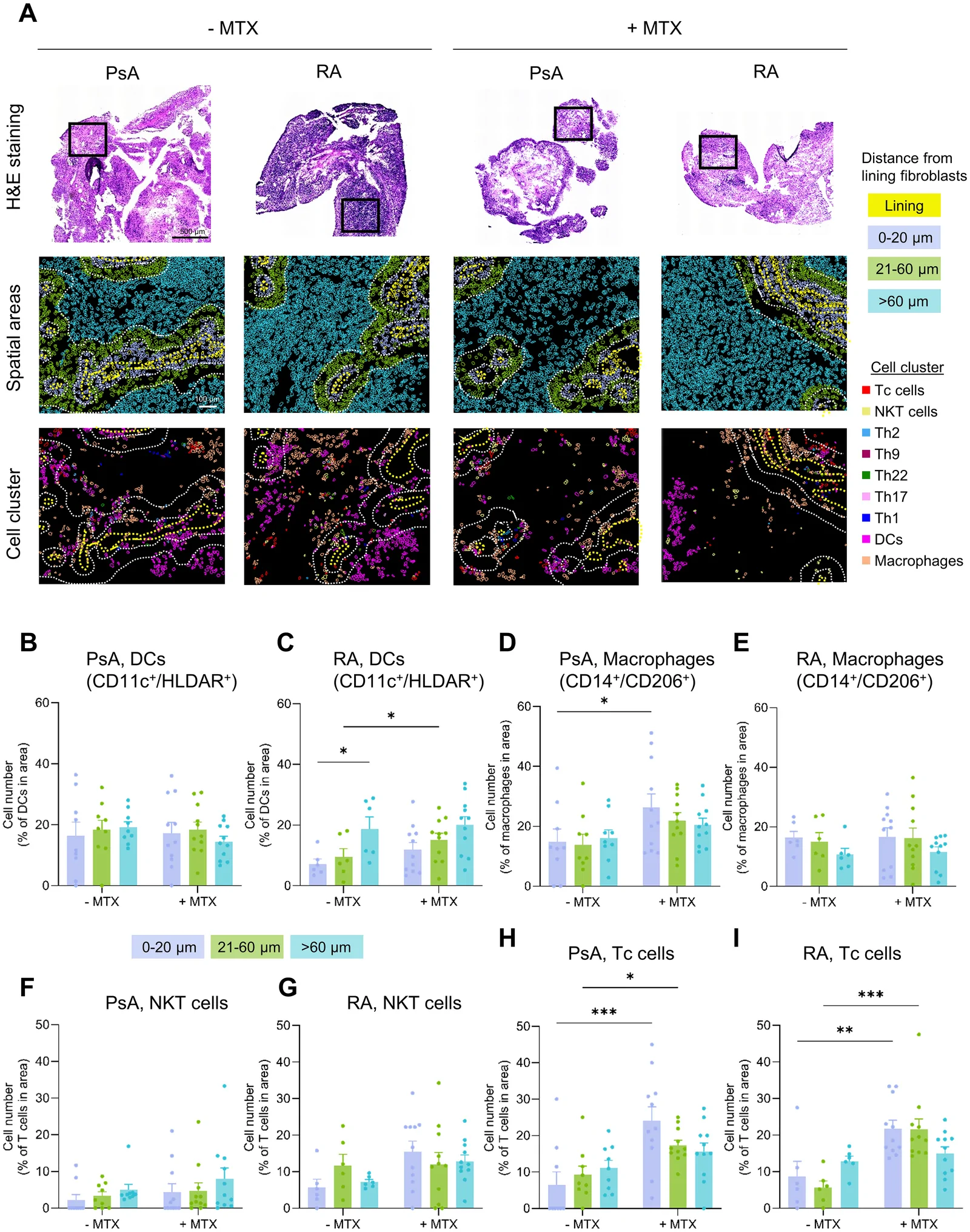
Distance analysis of synovial tissue. **(A)** Spatial distribution of immune cells relative to lining fibroblasts in synovial tissue, color-coded by distance: purple (0-20 µm), green (21-60 µm), blue (>60 µm). Area annotation is based on CD55 staining. **(B–I)** Percentages of DC2s **(B, C)**, CD14^+^/CD206^+^ macrophages **(D, E)**, NKT cells **(F, G)** and cytotoxic T cells **(H, I)** of all immune cells in 0-20 µm, 21-60 µm, and >60 µm distance to lining fibroblasts. Data are shown as mean of N = 6-11. Two-way ANOVA with *post-hoc* Tukey multiple comparison test, *p<0.05, **p<0.01, ***p<0.001.

More importantly, the Th cell populations demonstrated highly refined disease- and treatment-specific distribution patterns, resulting in area-specific distribution of immune responses (**Figure 6A**). Here, Th1 cells had the highest cell numbers in untreated PsA in the direct neighborhood (0-20 µm) of lining fibroblasts with a decreased number in 21-60 µm and being nearly absent in >60 µm (**Figure 6B**). In contrast, in RA they were absent in 0-20 µm while their cell number increased in the two other areas (**Figure 6C**). Interestingly, Th2 cells were mainly located in 20-60 µm distance in PsA (**Figure 6D**) while having very low densities in all three areas in RA (**Figure 6E**). Notably, in untreated PsA patients, the Th9 cells had the highest cell density in 21-60 µm distance to lining fibroblasts as seen for Th2 cells (**Supplementary Figures S6A, B**) whereas Th17 and Th22 had the highest cell density in in 0-20 µm distance as seen for Th1 cells (**Supplementary Figures S6C–F**).

**Figure 6:**
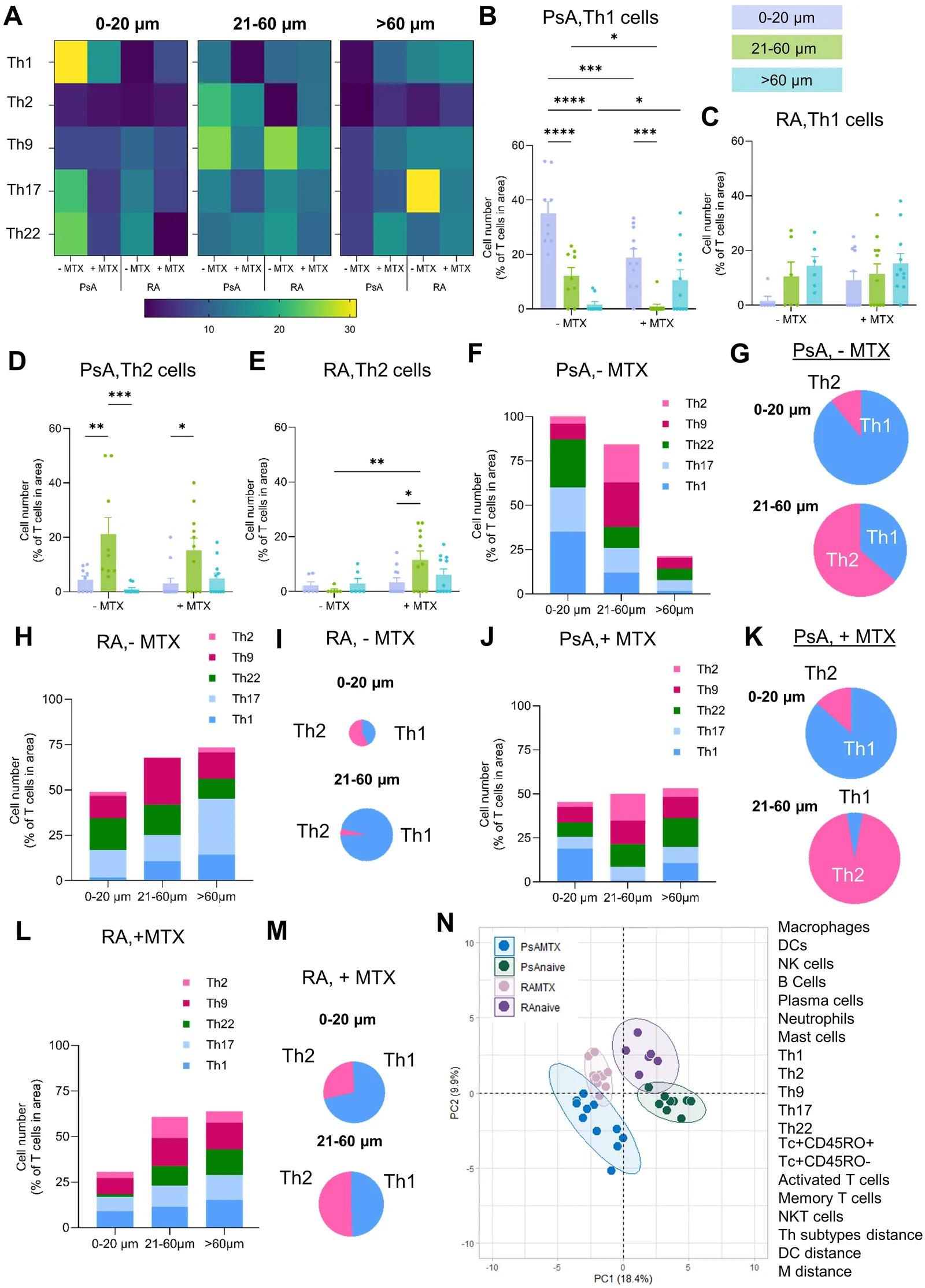
Spatial distribution of Th cells subsets in synovial tissue samples of PsA and RA patients. **(A)** Heatmap of mean cell frequency of Th cells subsets in synovial tissues, with color scale from 0 (blue, low frequency) to 30 (yellow, high frequency). **(B–E)** Percentages of Th1 **(B, C)**, Th2 **(D, E)**, in untreated and treated with MTX PsA and RA patients in 0-20 µm, 21-60 µm, and >60 µm distance to lining fibroblasts. Data are shown as mean of N = 6-11. Two-way ANOVA with *post-hoc* Tukey multiple comparison test, *p<0.05, **p<0.01, ***p<0.001, ****p<0.0001. **(F-I)** Percentages of Th subsets in untreated PsA **(F)** and RA **(H)** and ratio of Th1 and Th2 cells of untreated PsA **(G)** and RA **(I)** in the indicated distances to lining fibroblasts. Data are mean of N = 9-11. **(K–M)** Same as panels **(F–I)** except that data for MTX-treated patients are shown. Data are shown as mean of N = 6-11. **(N)** Principal component analysis based on the indicated immune cell numbers and their localization pattern based on the distance to lining fibroblasts in untreated PsA (N = 9) and RA (N = 6) patients, as well as PsA (N = 11) and RA (N = 11) patients receiving MTX treatment.

The responses of area-specific T cells become more apparent when T cell populations are plotted against their cell density per unit area. In untreated PsA, Th1, Th17 and Th22 cells dominate the direct neighborhood in 0-20 µm distance of the lining fibroblasts (**Figure 6F**) whereas Th2 and Th9 are more abundant in 21-60 µm distance (**Figure 6F**). Thus, in the direct neighborhood of the lining fibroblasts a niche with a high Th1/Th2 ratio was seen whereas this ratio switches towards an Th2 response in the adjacent area (**Figure 6G**). This separation of Th1 and Th2 responses was not observed in the corresponding RA synovium (**Figure 6H**). Here, Th1 and Th2 cells were both absent in 0-20 µm distance to the lining fibroblasts and a comparable modest presence of Th1 cells in the other areas (**Figures 6H, I**). Overall, the Th cell populations with the highest cell numbers in all three areas were Th9, Th17 and Th22 (**Figure 6H**). Synovial tissue of MTX-treated PsA showed 50% lower Th cell numbers in 0-20 µm distance to the lining fibroblasts (**Figure 6J**). Additionally, a significant lower cell density for Th1, Th17 and Th22 cells was seen (**Figure 6J**) with different Th1/Th2 ratios in both areas towards higher Th2 responses (**Figure 6K**). Conversely, synovial tissue of MTX-treated RA showed low number of total Th cells in the direct neighborhood of lining fibroblasts mostly of Th22 cells (**Figures 6L, M**).

Thus, in PsA a Th1 response dominates the immediate neighborhood of the lining fibroblasts, which borders an area where a Th2 response predominates. In RA Th1 and Th2 have very low cell densities in proximity to the lining fibroblasts. MTX-treated synovium showed lower Th cell density in PsA near the lining fibroblasts without dramatic changes in the immune cell distribution. Unlike PsA, synovial tissue of MTX-treated RA patients showed higher cell densities for Th1 and Th2 in these areas. Combining the quantitative data with the localization data of the various immune cell types allowed a better distinction of the disease and especially the MTX treatment groups in the principal component analysis (**Figure 6N**; **Supplementary Figure S7**).

## 4 Discussion

T cell phenotypes contribute significantly to disease pathogenesis in PsA and RA. Initial studies reported that PsA exhibits polyclonal CD8^+^ T cells expansion and RA a predominant CD4^+^ T cell profile (40, 41). More recently, the heterogeneity of T cell phenotypes in the two diseases has been studied in more detail (42–44), with a predominance of Th17 and Th22 in PsA (45–47). Also, some studies suggest that differential Fibroblast-like synoviocytes (FLS) and T cell subsets abundance could drive distinct pathogenesis in PsA and RA (34).

Although scRNA-seq analysis can capture T cells states, such as proliferating and regulatory, it is unable to characterize the classical T cell effector subsets, including Th1, Th2 and Th17 cells, in synovial tissue samples (33). Previous studies describing the spatial distribution of immune cells in the synovium of patients with PsA and RA showed that immunological niches are formed based on the identification of specific subpopulations of sublining fibroblasts (29–31, 34). By using spatial proteomics, this study is the first to comprehensively characterize the immune architecture of the cellular infiltrate and the number and distribution of myeloid cells, T cells and Th cell subtypes in the synovial tissue of patients with PsA and RA, both in untreated and in those treated with MTX, with the aim of identifying changes shared to both conditions or specific to each, as well as exploring the differences among disease stages and cross-sectionally evaluate the MTX-dependent therapeutic effect.

Here we showed that, also in the neighborhood of lining fibroblasts, distinct immunological niches can be observed. These niches exhibit disease and treatment-specific immune cell patterns, which are especially strong in the distribution of Th cell subpopulations. We found that the direct neighborhood of lining fibroblasts in PsA patients exhibits a predominant Th1/Th17 response. In addition, the presence of DC2 cells in this area in PsA was nearly doubling the number seen in RA patients, reflecting a classic immune response. In contrast, in RA, Th1 and Th2 cells were virtually absent and the number of dendritic cells was greatly reduced in the vicinity of the lining fibroblasts, which, in contrast, contained Th9, Th17 and Th22 cells, clearly pointing to a barrier-centered immune response, with a local microenvironment of immune cells and cytokines that differs markedly between PsA and RA. Accordingly, it could be hypothesized that the disease-specific differences between the direct neighborhoods of lining fibroblasts might help to explain why IL-23/IL-17 cytokine-axis blockage has such a strong efficacy in PsA compared to RA. At the joint level, dendritic cells release IL-23, which induces the differentiation and stabilization of Th17 cells and the secretion of IL-17 (48). Then IL-17 stimulates the production of other pro-inflammatory cytokines by various cell types, including synovial fibroblasts (2, 49). In this model, fibroblasts sustain IL-23 production and Th17 cell responses by dendritic cells (50).

A second interesting immunological niche was bordering the Th1/Th17 dominated direct neighborhood of lining fibroblasts in PsA, located in 21-60 µm distance of the lining fibroblasts, which was dominated by Th2/Th9 cells. While Th2 responses can counterbalance and suppress proinflammatory Th1/Th17-mediated responses (51), they can become pro-inflammatory when dysregulated. Th2/Th9 responses drive and amplify allergic/type 2 inflammation, IgE‐mediated responses, e.g. through IL-4/Il-13 (19). Therefore, the functional reason for the neighboring localization of the two very different immune responses is unclear and remains to be elucidated.

In accordance with previous published inhibitory effects of MTX on Th cell (52, 53), the number of Th cells decreased in the direct neighborhood of lining fibroblasts in both diseases by around 50%. Interestingly, in PsA the Th1/Th2 ratio was retained in the two immunological niches with Th1/Th17 dominating the direct neighborhood and Th2/Th9 the bordering area. Treatment with MTX had a clear effect on the number of immune cells and their distribution, even though these patients continued to show local signs of inflammation. Although conclusions regarding the effects of MTX treatment on synovial tissue should be interpreted with caution, the results clearly show substantial changes in the number and distribution of Th-cell subtypes (Th1-Th17, Th2-Th9) around the lining fibroblasts, suggesting interference in the interactions between fibroblasts, T cells and antigen-presenting cells in PsA synovial tissue.

In accordance with other publications (11, 54, 55), our systematic study, found no differences in the overall number of endothelial cells, sublining fibroblasts, or lining fibroblasts, or between PsA and RA patients without MTX treatment. However, other studies have reported less lining layer hyperplasia and a greater number of blood vessels in PsA patients (7, 56). The reason for these discrepancies is unclear, but it may be due to differences in the cohorts, such as synovitis scores, current treatments, and disease duration. Also, no differences in patients without MTX treatment were found in our study regarding the number of CD8 T cells and macrophages. However, other relevant findings showing disease-specific differences between PsA and RA are the higher number of dendritic cells in untreated RA vs PsA that become similar after MTX treatment as well as multiple differences in the number and spatial distribution of Th subpopulations untreated RA and PsA.

Our study has some limitations. First, the relative sample size could limit the power to detect subtle biological differences and may affect the generalizability of the findings. In addition, the cross-sectional design and lack of longitudinal sampling preclude a definitive assessment of disease-specific effects of MTX. In conclusion, our study demonstrates for the first time key differences between synovitis in RA and PsA, in which Th1-Th17 cells and dendritic cells are distributed very close to the lining fibroblasts in PsA, whereas in RA there is a completely different cellular environment, reminiscent of a barrier-centered immune response.

## Data Availability

The raw data supporting the conclusions of this article will be made available by the authors, without undue reservation.
