# Supplementary material for: T cells form distinct spatially restricted immune responses in synovium of patients with psoriasis arthritis and rheumatoid arthritis
Source: Front Immunol. 2026 Sep 30;17:1946638. doi: 10.3389/fimmu.2026.1946638 (PMC13661973; doi:10.3389/fimmu.2026.1946638)
Supplement: Supplementary file 1 [file DataSheet1.pdf]

Supplemental data S1

Table S1: Antibodies used for high content imaging

MACSima cycle	Fluorochrome	Antigen	Clone	Vendor	Order No.
1	APC	CD206	19.2	BD BioSciences	550889
	PE	CD8	REA734	Miltenyi Biotech	130-110-678
2	APC	CD7	M-T701	BD BioSciences	561604
	FITC	CD4	REA623	Miltenyi Biotech	130-114-531
	PE	CD1c	L161	BioLegend	331505
3	APC				
	FITC	CD11b	Bear1	Beckman	IM0530
	PE	CD3	REA613	Miltenyi Biotech	130-113-139
4	APC	CD161	REA631	Miltenyi Biotech	130-113-595
	FITC	CD19	SJ25C1	BioLegend	363008
	PE	CD27	REA499	Miltenyi Biotech	130-113-640
5	APC				
	FITC	CD45-RO	UCHL1	BioRad	MCA461F
	PE	CD11c	REA618	Miltenyi Biotech	130-121-314
6	APC	CD123	REA918	Miltenyi Biotech	130-115-265
	FITC	CD14	HCD14	BioLegend	325604
	PE	CD101	BB27	Miltenyi Biotech	130-095-225
7	APC				
	FITC	CD66b	G10F5	BioLegend	305104
	PE	CD55	REA1231	Miltenyi Biotech	130-124-489
8	APC	CD56	HCD56	BioLegend	318310
	FITC	CD31	WM59	BioRad	MCA1738F
	PE	CD127	A019D5	BioLegend	351312
9	APC				
	FITC	CXCR5-CD185	J252D4	BioLegend	356914
	PE	CD107a	H4A3	Miltenyi Biotech	130-119-872
10	APC	HLA-DR	AC122	Miltenyi Biotech	130-113-398
	FITC	CD52	YTH34.5	BioRad	MCA1642F
	PE	CCR4-CD194	205410	R&Dsystems	FAB1567P-100
11	APC				
	FITC	CD45-RA	REA562	Miltenyi Biotech	130-113-365
	PE	CD90	DG3	Miltenyi Biotech	130-117-388
12	APC	CD16	3G8	BD BioSciences	561248
	FITC				
	PE	CD163	GHI/61	BD Biosciences	556018
13	APC				
	FITC	CCR7-CD197	G043H7	BioLegend	353216
	PE	CCR3-CD193	5E8	BioLegend	310706
14	APC				
	FITC	Vimentin	REA409	Miltenyi Biotech	130-116-508
	PE	CD54	HA58	Invitrogen	12-0549-42
15	APC	CD117	REA787	Miltenyi Biotech	130-111-593
	FITC				
	PE	CD64	REA978	Miltenyi Biotech	130-116-196
16	APC				
	FITC	CD72	3F3	BioLegend	316204
	PE	CXCR4-CD184	12G5	BioLegend	306505
17	APC	CD62P	A4	BioLegend	304910
	FITC	CD62L	FMC46	BioRad	MCA1076F
	PE	CCR5-CD195	HEK/1/85a	BioLegend	313707
18	APC				
	FITC	CD138	REA929	Miltenyi Biotech	130-115-478
	PE	CCR6-CD196	REA190	Miltenyi Biotech	130-120-458
19	APC	CXCR3-CD183	G025H7	BioLegend	353708
	FITC				
	PE	CD38	REA671	Miltenyi Biotech	130-117-717
20	APC				
	FITC	MPO	REA491	Miltenyi Biotech	130-120-241
	PE	Ki67	REA183	Miltenyi Biotech	130-120-417
		DAPI	-	Sigma-Aldrich	D9542-10MG

Supplementary data S2:

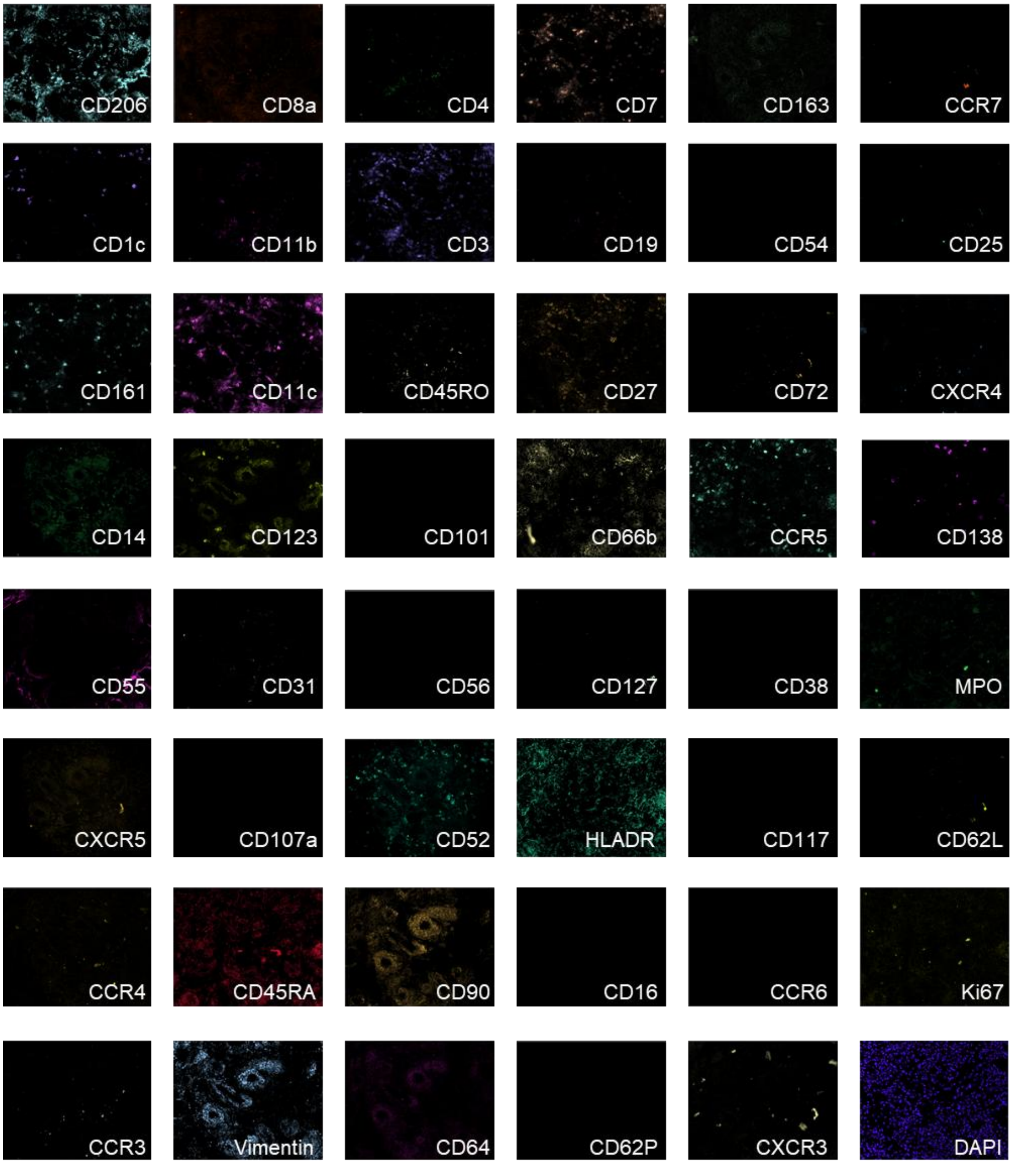

Figure S2: Immunofluorescence staining images of synovial tissue sample. Representative immunofluorescence staining images for immune markers in synovial tissue of a patient with PsA with MTX treatment, including markers used for analysis.

Supplementary data S3:

Krenn-Synovialitis-Score (KSS) of patients with PsA and RA

	PsA<br>- MTX	PsA<br>+ MTX	RA<br>- MTX	RA<br>+ MTX
**N**	8	8	3	9
**KSS**	3.88	3.13	6.33	4.67
**Lining**	1.25	1.0	2.67	1.78
**Stromal**	1.13	1.13	1.67	1.44
**Inflammatory Infiltrate**	1.5	1.13	2.0	1.44

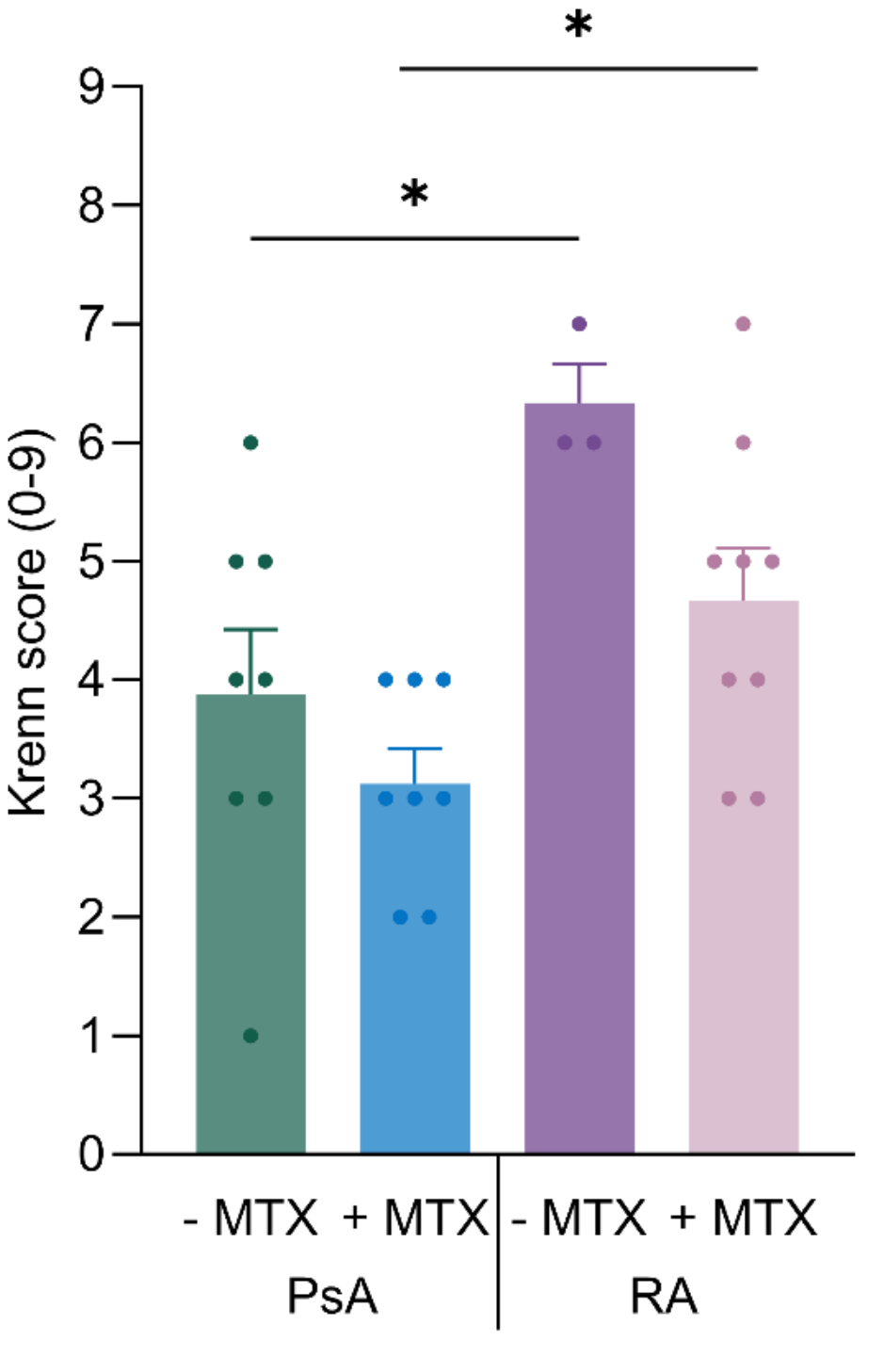

Figure S3: Krenn-Synovialitis-Scores (KSS) of synovial tissue samples from patients with PsA and RA. The upper table shows the detailed KSSs of the patients with PsA and RA. The Histogramm shows the distribution of the final KSSs for the four patient groups. Data are shown as mean of N = 3-9. Mann-Whitney test, *p<0.05.

Supplementary data S4:

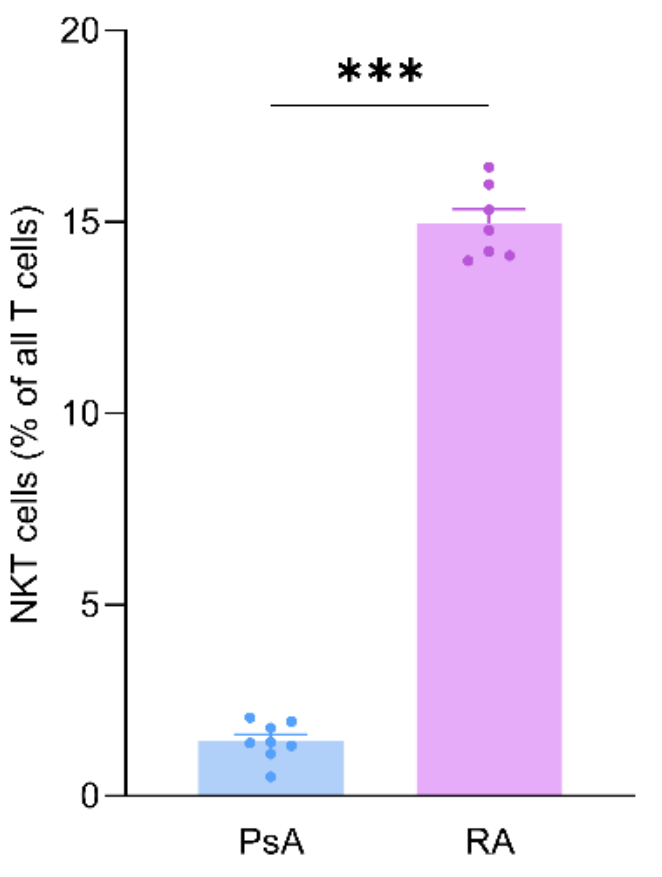

Figure S4: Low-grade synovial inflammation in RA patients treated with MTX is enriched of NKT cells compared to PsA patients treated with MTX. Percentages of NKT cells among total T cells in synovial tissues from PsA and RA patients treated with MTX. Data are shown as mean of N = 7-8, Mann Whitney test, ***p<0.001

Supplementary data 5:

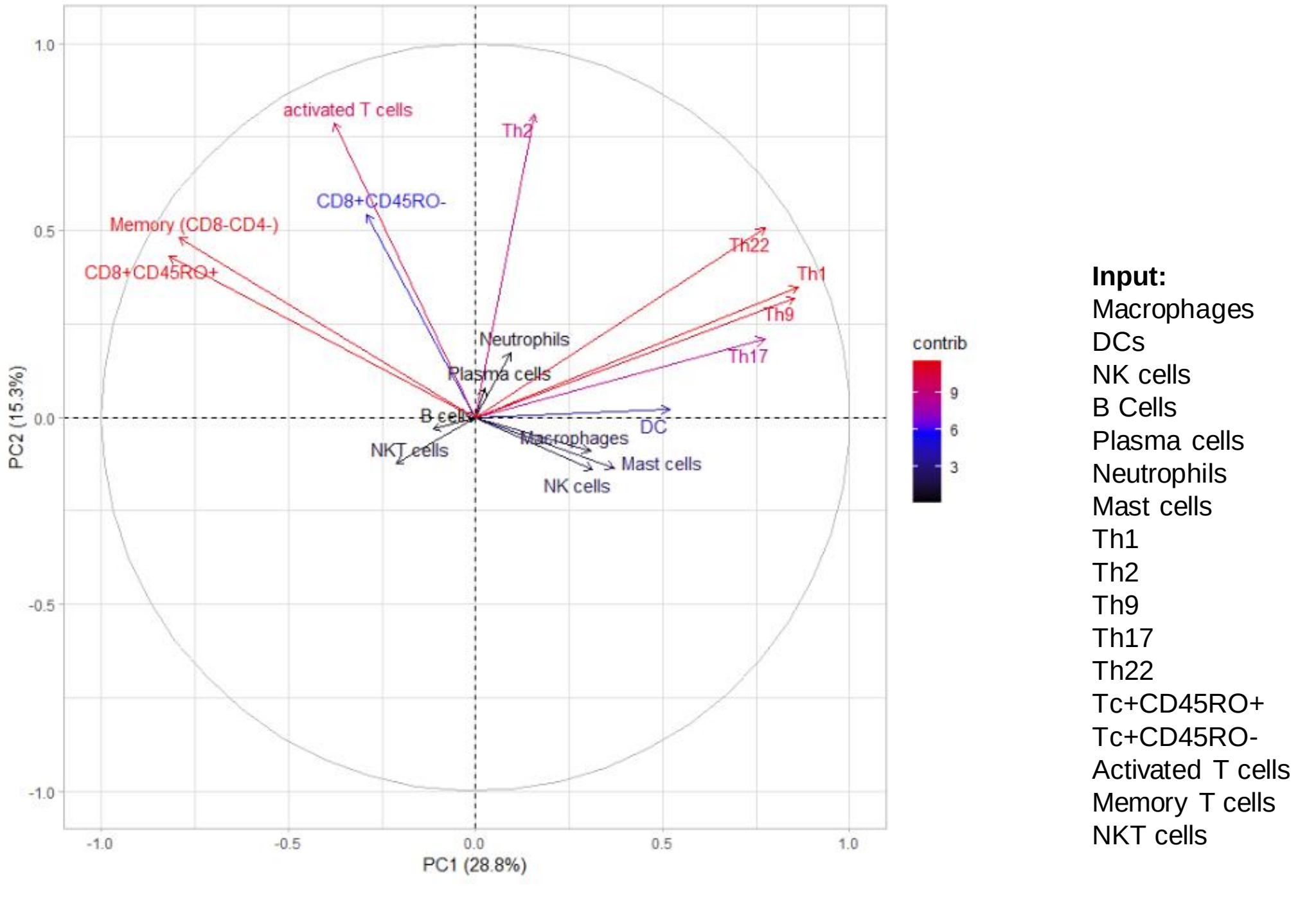

Figure S5: Principal component analysis based on the MACSima analysis differentiates between untreated PsA and RA patients. Contribution of the indicated cell type clusters to principal component analysis to the principal component analysis shown in Figure 2A.

Supplementary data S6:

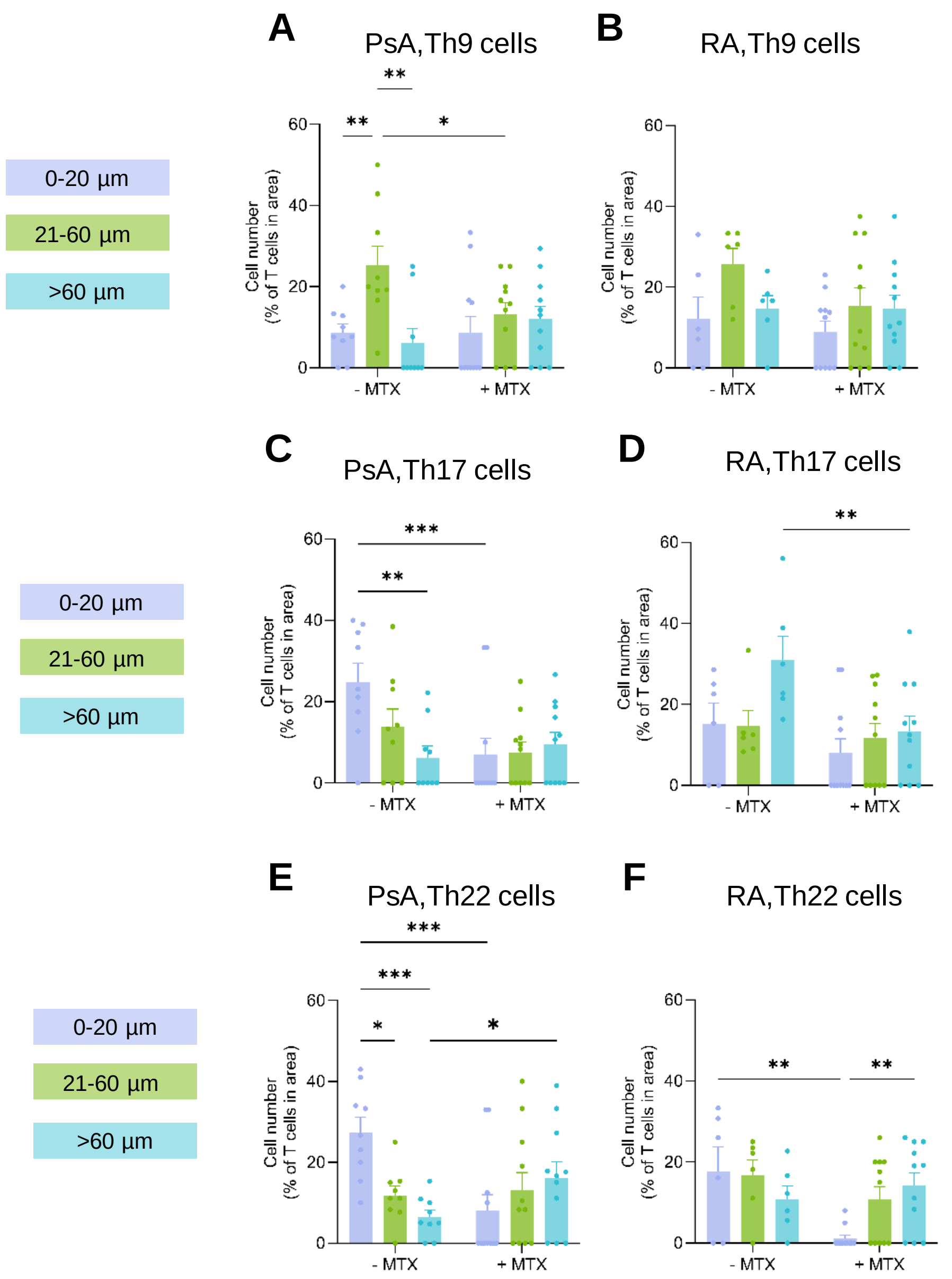

Figure S6: Spatial distribution of Th cells subsets in synovial tissue samples of PsA and RA patients. (A-F) Percentages of Th9 (A, B), Th17 (C, D) and Th22 (E, F) in untreated and treated with MTX PsA and RA patients in 0-20 µm, 21-60 µm, and >60 µm distance to lining fibroblasts. Data are shown as mean of N = 6-11. Two-way ANOVA with post-hoc Tukey multiple comparison test, *p<0.05, **p<0.01, ***p<0.001.

Supplementary data S7:

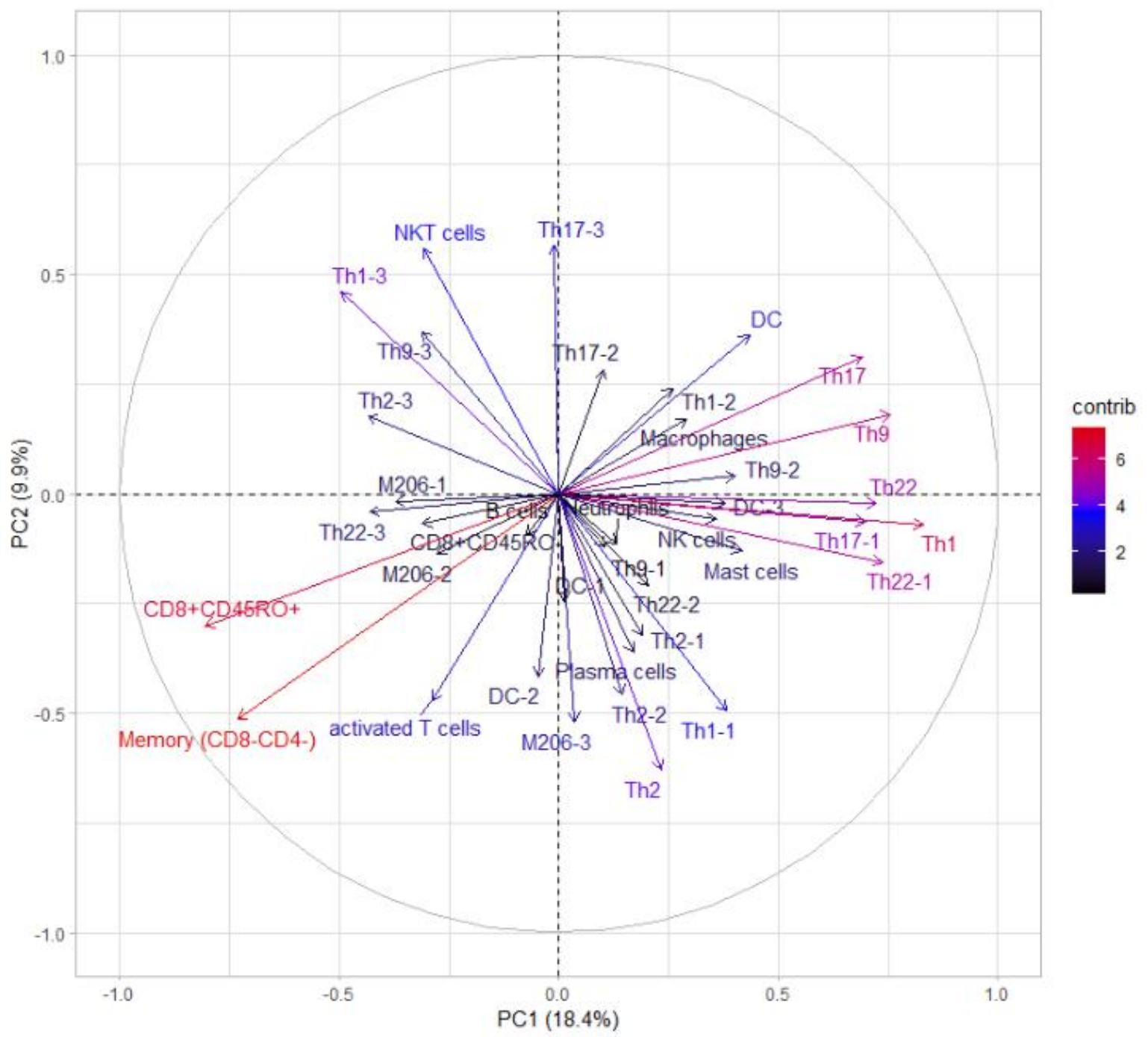

Input:
Macrophages
DCs
NK cells
B Cells
Plasma cells
Neutrophils
Mast cells
Th1
Th2
Th9
Th17
Th22
Tc+CD45RO+
Tc+CD45RO-
Activated T cells
Memory T cells
NKT cells
Th subtypes distance
DC distance
M distance

Figure S7: Principal component analysis based on the MACSima analysis differentiates between PsA and RA patients with or without MTX treatment. Contribution of the indicated cell type clusters to principal component analysis to the principal component analysis shown in panel A.
